# Robotic mitral repair after Rastelli operation and replacement of the aortic valve and right ventricle–pulmonary artery conduit

**DOI:** 10.1016/j.xjtc.2023.09.007

**Published:** 2023-09-15

**Authors:** Makoto Hibino, Douglas A. Murphy, Amalia A. Jonsson, Michael E. Halkos

**Affiliations:** Division of Cardiothoracic Surgery, Department of Surgery, Emory University School of Medicine, Atlanta, Ga

**Figure:**
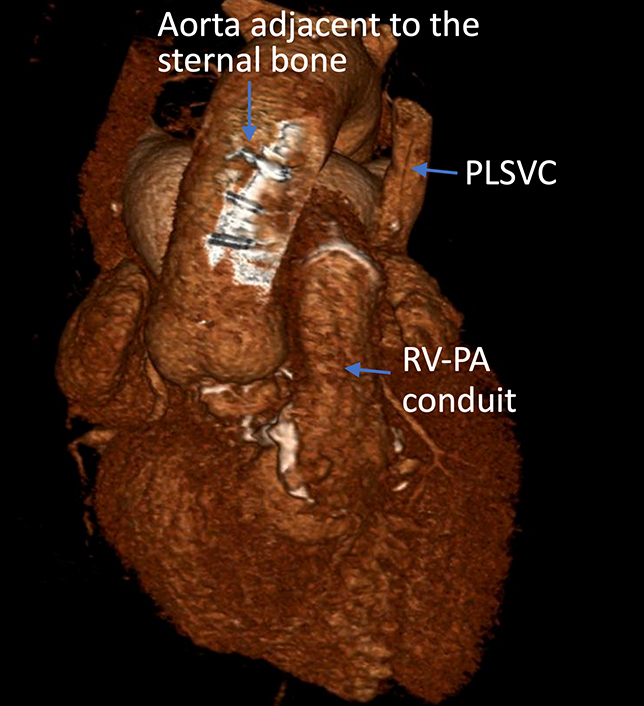
Three-dimensional reconstruction of computed tomography scan.

**Central Message**

The robotic approach offers a less-invasive and effective alternative to traditional redo-sternotomy or right thoracotomy in a patient who has undergone a Rastelli operation.

Mitral surgery is a common procedure in adults after initial repair for congenital cardiac disease^1^; however, redo operations can be challenging. We have used a lateral endoscopic approach with robotics (LEAR) technique for redo cases to repair mitral regurgitation (MR) to promote better visualization, effective operative manipulation, and less invasiveness by avoiding redo sternotomy.^2^

## Case Summary

A 37-year-old man developed symptomatic severe MR after an initial Rastelli operation for D-transposition of the great arteries with ventricular septal defect and subpulmonic stenosis at 4 year old and subsequent 25-mm bioprosthetic aortic valve replacement and right ventricle–pulmonary artery conduit replacement (23-mm bioprosthetic valve and 24-mm Dacron tube) for infective endocarditis at 27 year old. This study was approved by the institutional review board (IRB00073906, April 21, 2014); written informed consent for publication of study data was obtained from the patient.

**Figure 1:**
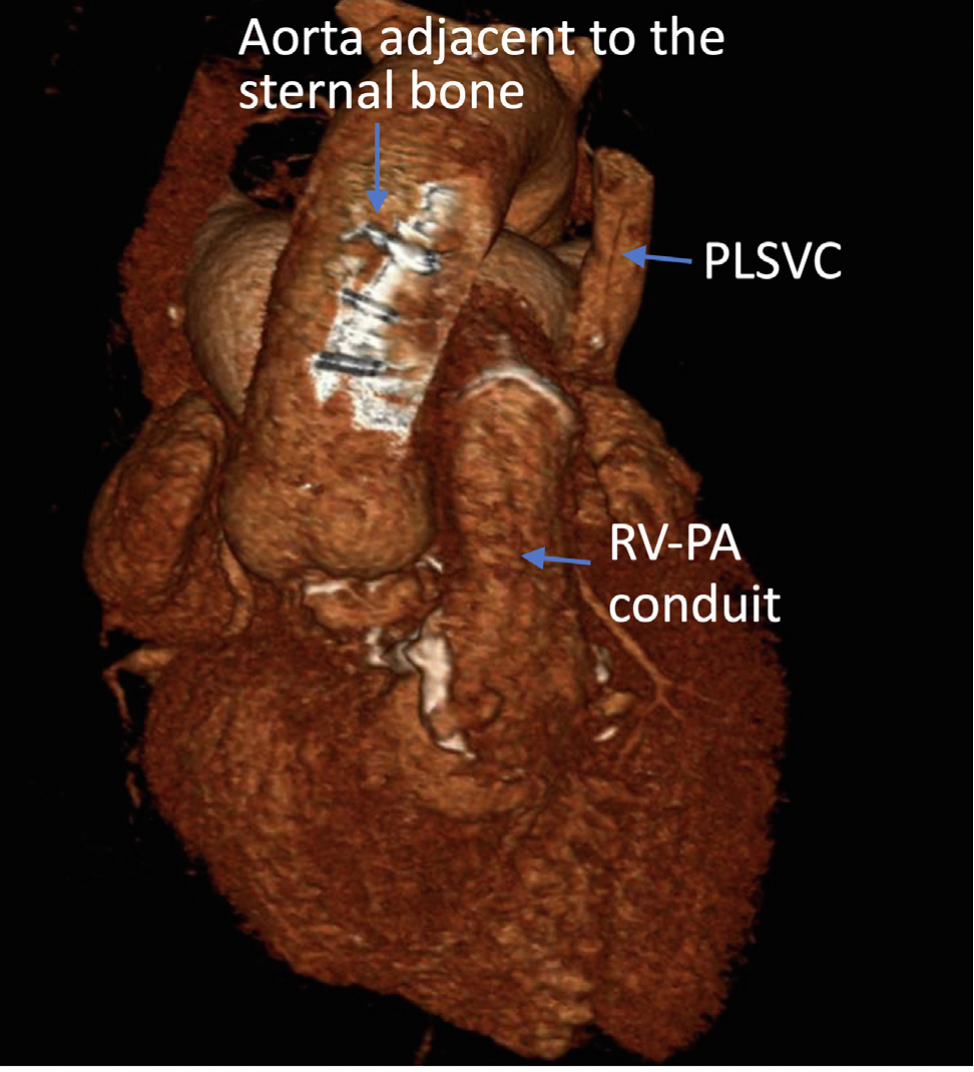
Three-dimensional reconstruction of computed tomography scan. The aorta was adjacent to the sternal bone. *PLSVC*, Persistent left superior vena cava; *RV-PA*, right ventricle-pulmonary artery.

**Figure 2:**
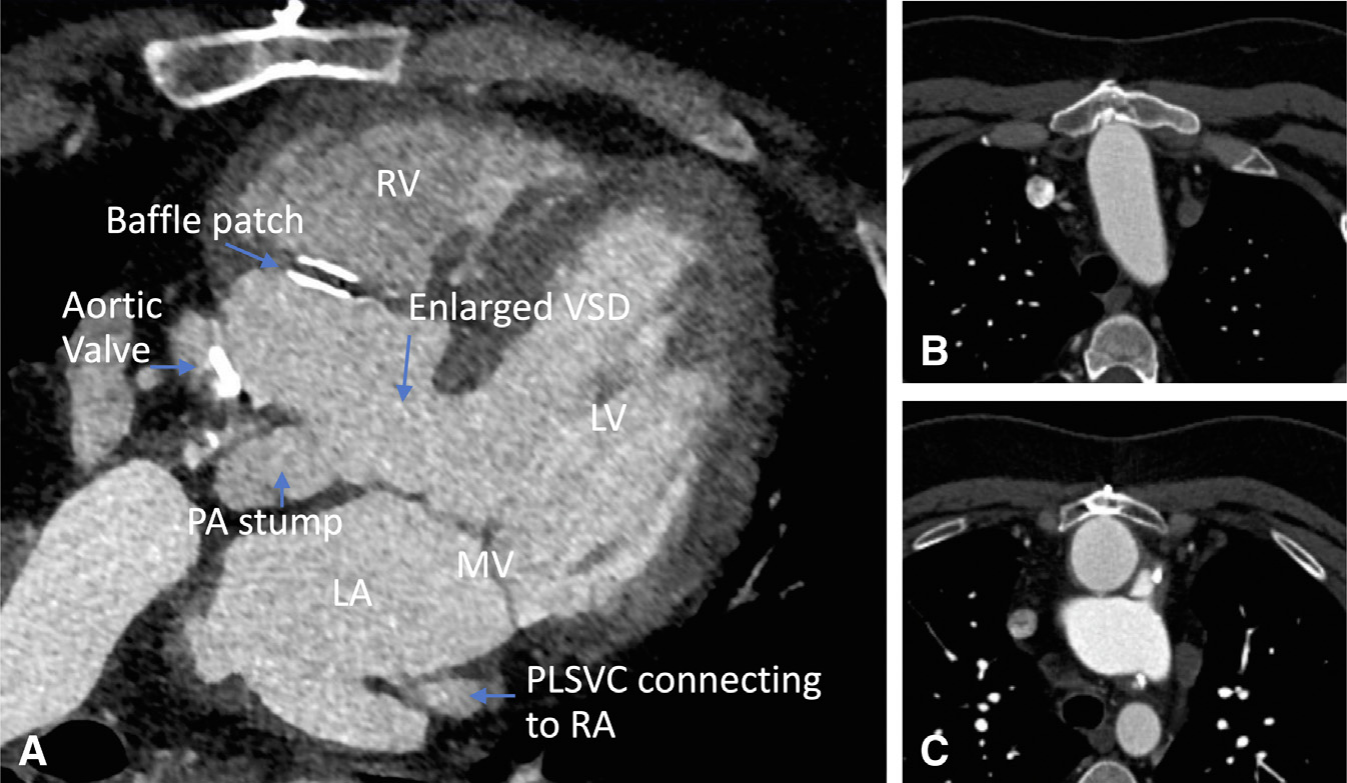
Preoperative computed tomography scan in axial view. A, Persistent left superior vena cava connected to the right atrium. The mitral valve was located just behind the pulmonary artery stump. B and C, The aorta was adjacent to the sternal bone. *RV*, Right ventricle; *VSD*, ventricular septal defect; *LV*, left ventricle; *MV*, mitral valve; *LA*, left atrium; *PLSVC*, persistent left superior vena cava; *RA*, right atrium.

**Figure 3:**
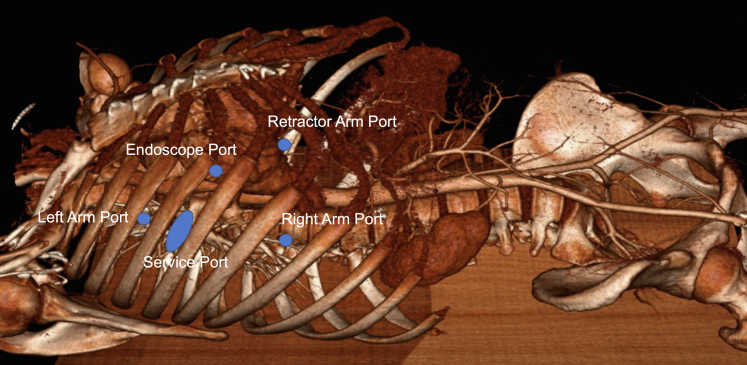
Our standard port placements in a lateral endoscopic approach with robotics technique. The femoral arteries were narrow and bilateral cannulation was required for perfusion and delivery of intra-aortic occlusion device.

Preoperative echocardiography showed flail P2 leaflet. Preoperative computed tomography showed persistent left superior vena cava, the aorta adjacent to the sternal bone, and the mitral valve located just behind the pulmonary artery stump (Figures 1 and 2). We employed the LEAR technique to avoid a third sternotomy (Figure 3). We cannulated the bilateral internal jugular veins, left femoral vein, and right femoral artery to establish cardiopulmonary bypass and the left femoral artery to deliver an IntraClude intra-aortic occlusion device (Edwards Lifesciences). We carefully dissected the adhesions around the left atrium. After cardiac arrest using the occlusion device, we opened the left atrium and exposed the mitral valve. Severely dilated mitral annulus with P2 prolapse was identified. We resected the P2 segment and closed the cleft between P1 and P2. We placed a 34-mm SimuPlus annuloplasty band (Medtronic) to the dilated annulus. As the coaptation depth was still shallow, we applied a A2-P2 edge-to-edge stitch. The operative time, cardiopulmonary bypass time, and crossclamp time were 483, 220, and 108 minutes, respectively. Postoperative echocardiography did not show residual MR, with mean pressure gradient of 2 mm Hg. Red blood cell transfusion was not required. The patient was extubated the following day and discharged home 5 days after surgery.

## Comments

Redo valve surgery carries a high risk of morbidity and mortality.^3^ In the present case, proximity of the aorta to the sternal bone amplifies the risks, compounding the standard challenges associated with redo surgery. Consequently, we opted for a robotic approach to address mitral valve repair. In addition, through the use of a robotic approach, we achieved sufficient visualization from a lateral angle without the need for extensive dissection of adhesion or distortion of the heart. For the purpose of achieving optimal decompression of the right atrium and enhancing visualization of the mitral valve, we cannulated both the superior jugular veins.

In this case, we employed an intra-aortic occlusion device to occlude the ascending aorta, deliver cardioplegia solution, and vent blood and air from the aortic root. Using the intra-aortic occlusion device eliminated the necessity for dissection around the ascending aorta. The precise placement of the occlusion device plays a pivotal role in achieving effective occlusion and successful cardioplegia delivery (Video 1). Transesophageal echocardiography provides essential images for guiding the device's positioning during the balloon inflation. The use of indocyanine green within the balloon of the aortic occlusion device is also efficacious in a majority of redo cases if the lateral wall of the ascending aorta is exposed. Continuous monitoring of blood pressure in the right arm serves as a valuable method for promptly detecting significant migration. It is crucial to closely monitor aortic root pressure during mitral valve tests using pressurized saline solution to prevent the migration of the aortic occlusion device.

## Conclusions

We report a successful robotic approach for MR after 2 previous sternotomies including a Rastelli operation. The LEAR technique provided a less-invasive and effective alternative to traditional redo-redo sternotomy or right thoracotomy.

## Conflict of Interest Statement

Dr Murphy reported speaker for Intuitive Surgical. All other authors reported no conflicts of interest.

The *Journal* policy requires editors and reviewers to disclose conflicts of interest and to decline handling or reviewing manuscripts for which they may have a conflict of interest. The editors and reviewers of this article have no conflicts of interest.

## Supplementary Data

Video 1 Robotic approach for third redo mitral repair following Rastelli operation. Video available at: https://www.jtcvs.org/article/S2666-2507(23)00310-3/fulltext.
