# Supplementary figures and images for: Robotic mitral repair after Rastelli operation and replacement of the aortic valve and right ventricle–pulmonary artery conduit
Source: JTCVS Tech. 2023 Sep 15;22:96–8. doi: 10.1016/j.xjtc.2023.09.007 (PMC10750848; doi:10.1016/j.xjtc.2023.09.007)

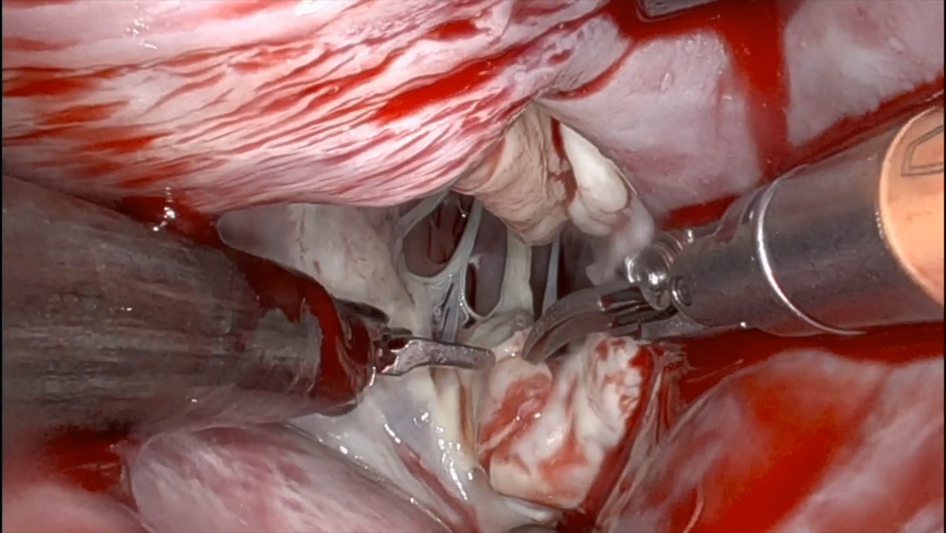

Supplement: Video 1 — Robotic approach for third redo mitral repair following Rastelli operation. Video available at: https://www.jtcvs.org/article/S2666-2507(23)00310-3/fulltext. [file fx2.jpg]
